# Zinc cooperates with p53 to inhibit the activity of mitochondrial aconitase through reactive oxygen species accumulation

**DOI:** 10.1002/cam4.2130

**Published:** 2019-04-10

**Authors:** Ya‐Nan Xue, Ya‐Nan Liu, Jing Su, Jiu‐Ling Li, Yao Wu, Rui Guo, Bing‐Bing Yu, Xiao‐Yu Yan, Li‐Chao Zhang, Lian‐Kun Sun, Yang Li

**Affiliations:** ^1^ Department of Pathophysiology, College of Basic Medical Sciences Jilin University Changchun, Jilin China

**Keywords:** mitochondrial aconitase, p53, ROS, zinc

## Abstract

Metabolic reprogramming is a central hallmark of cancer. Therefore, targeting metabolism may provide an effective strategy for identifying promising drug targets for cancer treatment. In prostate cancer, cells undergo metabolic transformation from zinc‐accumulating, citrate‐producing cells to citrate‐oxidizing malignant cells with lower zinc levels and higher mitochondrial aconitase (ACO2) activity. ACO2 is a Krebs cycle enzyme that converts citrate to isocitrate and is sensitive to reactive oxygen species (ROS)‐mediated damage. In this study, we found that the expression of ACO2 is positively correlated with the malignancy of prostate cancer. Both zinc and p53 can lead to an increase in ROS. ACO2 can be a target for remodeling metabolism by sensing changes in the ROS levels of prostate cancer. Our results indicate that targeting ACO2 through zinc and p53 can change prostate cancer metabolism, and thus provides a potential new therapeutic strategy for prostate cancer.

## 1 INTRODUCTION

Metabolic reprogramming is a central hallmark of cancer.^1^ Multiple studies have shown that the canceration and development of many cancers are accompanied by metabolic remodeling.^2^, ^3^ Normal prostate cells have the uniquely specialized function of accumulating and secreting extremely high levels of citrate, the main component of prostatic fluid.^5^, ^6^ The citrate production activity of prostate cells is achieved by cellular accumulation of high levels of zinc that inhibit citrate oxidation. Zinc has been shown to inhibit the activity of mitochondrial aconitase (ACO2), an enzyme that catalyzes the conversion of citrate to isocitrate, resulting in the accumulation of citrate that is unable to enter the Krebs cycle.^5^, ^7^ Additionally, prostate cancer cells do not exhibit the Warburg effect.^8^ In contrast, malignant cells have lower levels of zinc, which eliminates the inhibitory effect on ACO2, thus permitting the oxidation of citrate via a functional Krebs cycle with increased production of ATP.^6^ As a consequence, the cells are converted from citrate‐producing to citrate‐oxidizing cells. Mycielska et al demonstrated that the oxidation of citrate in PC‐3 human prostate cancer cells provides energy for proliferation and metastasis of the cells.^9^ These studies demonstrate that zinc and ACO2 are critical regulators of citrate metabolism in prostate cancer cells. However, the mechanism by which zinc inhibits ACO2 is not clear.

ACO2 is an iron‐sulfur dehydratase containing a tetrairon‐tetrasulfide (4Fe‐4S) cluster,^10^ which plays an important role in the maintenance of mitochondrial functions such as oxidative phosphorylation and energy production.^10^, ^11^ Because of the iron‐sulfur group, ACO2 is sensitive to reactive oxygen species (ROS), which can affect ACO2 structure and activity.^10^, ^14^, ^15^ A decrease of ACO2 activity has been observed in some neurodegenerative diseases and senescence related to oxidative stress.^10^, ^16^, ^17^ These studies suggest that redox state has a significant effect on the activity of ACO2. Recent studies have shown that compared with normal cells, cancer cells are under increased oxidative stress associated with oncogenic transformation, alterations in metabolism, and increased generation of ROS.^18^ However, because ROS are chemically active and can inflict severe cellular damage, the fact that cancer cells are under increased ROS stress may provide a unique opportunity to eliminate them.

The trace element zinc plays an important role in the regulation of cellular ROS levels. Provinciali found that zinc can induce apoptosis in melanoma cells by increasing ROS.^19^ Guo found that zinc can induce the production of hydroxyl radicals in rat retinal ganglion cells, leading to apoptosis.^20^ Therefore, we speculate that the ROS levels in cells induced by zinc may lead to inactivation of ACO2.

p53 is a zinc‐dependent metalloprotein. We previously found that zinc promoted apoptosis and cell cycle arrest induced by Pmp53 (a plasmid containing both MDM2 small interfering RNA and the wild‐type p53 gene).^21^, ^22^ Further research has shown that p53 also plays an important role in the regulation of redox state and metabolism in the tumor cells.^23^, ^24^ The SIRT3 deacetylase regulates the transcription and deacetylation of superoxide dismutase 2 (SOD2) to decrease ROS levels.^27^, ^28^ Guo found that activated p53 inhibits SOD2 expression, leading to increased ROS levels in hepatocellular carcinoma.^30^ Li found that p53 induces growth inhibition by modulating SIRT3 deacetylase activity in EJ‐p53 cells.^31^In addition, Abigail found that ACO2 and SOD2 can form a co‐regulated multi‐protein complex in mouse heart mitochondria.^32^ Therefore, these results suggest the possibility of a p53‐SIRT3‐SOD2‐ROS axis that may function in the regulation of ACO2.

Based on the above studies, we hypothesize that both zinc and p53 can lead to an increase in ROS levels, which can result in impaired ACO2 activity and affect cell metabolism. Thus, ACO2 may become both a target for sensing and responding to the ROS levels and a trigger for metabolic remodeling. Therefore, targeting ACO2 through zinc and p53 may provide a new strategy for the therapy of prostate cancer.

## 2 MATERIALS AND METHODS

### 2.1 Cell culture and reagents

The prostate cancer cell lines DU145 and PC3 were purchased from the American Type Culture Collection (ATCC) and cultured under conditions recommended by ATCC. Cell lines were authenticated using Short Tandem Repeat analysis as described in 2012 in ANSI Standard (ASN‐0002). Construction of the eukaryotic expression vector Pmp53 was previously reported.^33^ Anti‐p53 (sc‐126) and anti‐MDM2 (sc‐965) were purchased from Santa Cruz Biotechnology (Santa Cruz, CA); anti‐cleaved‐caspase 3 (9665s) and anti‐SIRT3 (2627s) were purchased from Cell Signaling Technology (Danvers, MA); anti‐actin (60008) was purchased from Proteintech (Rocky Hill, NJ); and anti‐ACO2 (ab110321) was purchased from Abcam (Cambridge, MA). Zinc was purchased from Sigma (St. Louis, MO). NAC, paclitaxel (PTX), and TPEN were purchased from MedChem Express (Monmouth Junction, NJ, USA). Transfections were performed using Lipofectamine 2000 (Invitrogen, Carlsbad, CA).

### 2.2 MTT cell viability assay

After seeded in 96‐well plates for 24 hours, cells were transfected. After 6 hours of transfection, cells were treated with zinc and PTX for 24 hours. This applies to all future instances. Media was removed, MTT (0.5 mg/mL) was added and cells were incubated for 4 hours. Formazan crystals were dissolved with 150 µL DMSO and absorbance was measured at a wavelength of 490 nm.

### 2.3 Western blot

Protein extracts were separated on an SDS‐PAGE gel and transferred to a nitrocellulose membrane. The membrane was blocked with nonfat milk and incubated with primary antibodies overnight. The membranes were then incubated with HRP‐conjugated IgG. Signals were detected with enhanced chemiluminescence.

### 2.4 Biochemical assays

Cells were transfected and treated with indicated drugs for 24 hours and then collected. Hydroxyl radicals were detected using a Hydroxyl Radical Assay Kit (Jiancheng, Nanjing, China), ATP was measured using an ATP assay kit (Beyotime Biotechnology, Shanghai, China), cell oxygen consumption rate (OCR) was measured using Mito‐Xpress and pH‐Xtra kits (Luxcel Bioscience, Cork, Ireland), mitochondria extraction from cultured cells was performed (MP‐007, Inventbiotech), ACO2 activity was determined using an Aconitase Activity Assay Kit (MAK051, Sigma), and isocitrate and citrate concentrations were determined using an Isocitrate Assay Kit (MAK319, Sigma) and a Citrate Assay Kit (MAK057, Sigma), respectively. All assays were performed according to the manufacturers' protocols.

### 2.5 Flow cytometry

The production of mitochondrial ROS was evaluated by staining with MitoSOX Red Mitochondrial Superoxide Indicator (Invitrogen) and apoptosis was determined by Annexin‐V and PI (556547, BD Biosciences). Both evaluations were performed according to the manufacturers' protocol. Cells were analyzed using a BD Accuri C6 flow cytometer (Becton Dickinson, Franklin Lakes, NJ).

### 2.6 Xenograft models

Male BALB/c nude mice (Beijing Vital River Laboratory Animal Technology, China) were housed in standard microisolator conditions free of pathogens and used in accordance with the Animal Care and Use protocol approved by Jilin University. Mice at 6‐8 weeks of age (n = 15) were subcutaneously injected with 5 × 10^7^ DU145 cells in the left flank. When tumors reached a mean size of 30 mm^3^, animals were randomly split into five groups (n = 3/group): control, PTX, Pmp53, zinc, and Pmp53 + zinc + PTX groups. PTX (10 mg/kg body weight) was administered intraperitoneally twice a week. Pmp53 plasmid formulated with attenuated *Salmonella typhi* Ty21a (1 × 10^7^ CFU/100 µL, total 100 µL) was injected intravenously every week. Zinc (10 mg/kg body weight) was administered every 2 days by gavage. After 20 days of treatment, mice were sacrificed and tumors were removed for analysis.

### 2.7 SIRT3 and ACO2 knockdown by siRNA

The SIRT3‐RNAi sequence was TCGATGGGCTTGAGAGAGT. The ACO2‐RNAi sequences were CCGCTACTACAAGAAACAT (siACO2‐91), TGCTAGAGAAGAACATTAA (siACO2‐12), and TGCCATTATGACCAACTAA (siACO2‐05). The nontarget siRNA sequence was TTCTCCGAACGTGTCACGT. The siRNAs were constructed by Genechem (Shanghai, China).

### 2.8 Tissue microarray and immunohistochemistry

Tissue microarray (TMA) was constructed in collaboration with Shanghai Biochip Company (Shanghai, China) using tumorous (T) and adjacent non‐tumorous (ANT) tissues from 90 prostate cancer patients (180 cores). Immunohistochemistry (IHC) staining for ACO2 (ab110321; Abcam) expression was performed on 5 µm sections of the TMA. TMA slides were scanned using the Aperio slide scanner and analyzed by ImageScope software (Aperio). IHC staining was scored by two independent pathologists using the following scoring: proportion of positive stain (0, 0%; 1, 1‐25%; 2, 26‐50%; 3, 51‐75%; 4, >75%) and mean stain intensity (0 for absence of staining, 1 for weak staining, 2 for moderate staining, and 3 for strong staining). Final scores were calculated as the product of the proportion of positive stained cells and the mean stained intensity (range 0‐12). Samples with scores 0‐5 were considered ACO2 negative, and samples with scores 6‐12 were ACO2 positive.

### 2.9 Statistical analysis

Results are expressed as mean values ± standard deviation. ANOVA was performed to compare results among the groups. All experiments were repeated three times. Chi‐squared analysis and Spearman rank correlation analysis were used to analyze the TMA data. Statistical analysis was performed using SPSS19.0 statistical software (SPSS Inc, Chicago, IL).

## 3 RESULTS

### 3.1 Expression of ACO2 positively correlates with prostate cancer malignancy and drug resistance

**Figure 1:**
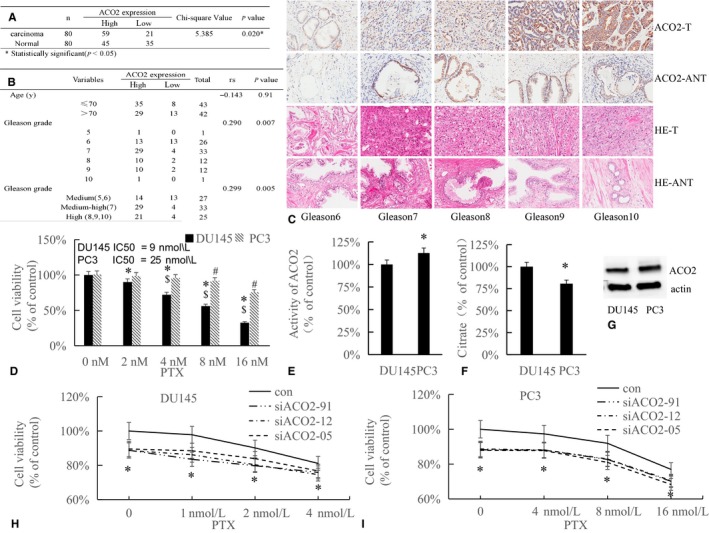
Expression of ACO2 positively correlates with prostate cancer malignancy and drug resistance. (A, B) Clinical characteristics and ACO2 expression in 80 prostate cancer tumorous (T) and matched adjacent non‐tumorous (ANT) tissues. (C) Immunohistochemical staining of ACO2 and hematoxylin and eosin staining in prostate T and ANT tissues (magnification, 400×). (D) DU145 and PC3 cells were treated with different doses of paclitaxel (PTX) for 24 hours. Cell viability was determined by MTT assay. Bars, SD (n = 3). *, #*P* < 0.01 vs control, $*P < *0.01 DU145 vs PC3 cells. (E, F) ACO2 activity and citrate levels in DU145 and PC3 cells were analyzed. Bars, SD (n = 3). *P < 0.01 PC3 vs DU145 cells. (G) Western blot analysis for the expression of ACO2 in DU145 and PC3 cells. (H, I) DU145 and PC3 cells transfected with siACO2 were treated with different doses of PTX. Cell viability was determined by MTT assay. Bars, SD (n = 3). *P < 0.01 siACO2s vs control

We first investigated the protein expression of ACO2 in a TMA with prostate cancer T and ANT tissue samples from 90 prostate cancer patients. We found that the positive expression of ACO2 in T tissues was significantly higher than in ANT tissues (Figure 1A,C). The expression of ACO2 also positively correlated with prostate cancer malignancy (Figure 1B,C), while ACO2 expression was independent of age (Figure 1B).

In cancer, high malignancy is often accompanied by chemotherapy resistance. And ACO2 is positively correlated with malignancy. We then examined the sensitivity of prostate cancer DU145 and PC3 cells to PTX, as well as the protein expression and activity of ACO2 and citrate levels. We found that DU145 cells were more sensitive to PTX than PC3 cells (Figure 1D). ACO2 expression and activity were decreased in PTX‐sensitive DU145 cells compared with PTX‐insensitive PC3 cells (Figure 1E,G), along with an increase in citrate (Figure 1F). This result indicated that ACO2 was decreased in prostate cancer cells with PTX sensitivity. To confirm this possibility, we transfected both DU145 and PC3 cells with siACO2, treated cells with different does of PTX, and examined cell viability. The results showed that DU145 and PC3 cells transfected with siACO2 showed increased sensitivity to PTX (Figure 1H,I). Together, these results suggest that expression and activity of ACO2 positively correlates with prostate cancer malignancy and may be involved in drug resistance.

### 3.2 Zinc combined with p53 increases the chemosensitivity of prostate cancer cells with significantly decreased ACO2 activity

**Figure 2:**
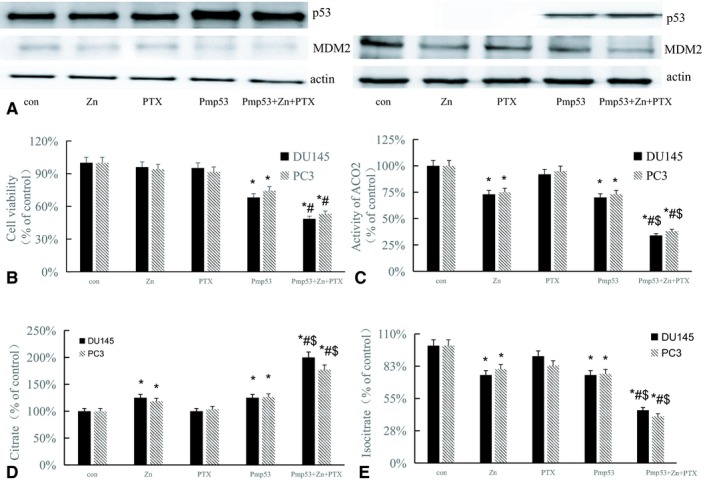
Zinc combined with p53 increases the chemosensitivity of prostate cancer cells and decreases ACO2 activity. DU145 and PC3 cells were treated with zinc or paclitaxel (PTX) and/or transfected with Pmp53 or pcDNA3.1 vector plasmid (as control group). (A) Western blot analysis was performed for the expression of p53 and MDM2. (B) Cell viability was determined by MTT assay. Bars, SD (n = 3). *P < 0.01 vs control, #*P* < 0.01 vs Pmp53. ACO2 activity (C), citrate (D) and isocitrate levels (E) were examined. Bars, SD (n = 3). *P < 0.01 vs control, #*P < *0.01 vs Zn, $*P < *0.01 vs Pmp53

Our previous study found that Pmp53 could increase the sensitivity of ovarian cancer to cisplatin.^22^ Here, we treated DU145 and PC3 cells with zinc, or PTX and/or Pmp53, the western blot results showed that after transfected with Pmp53, the expression of p53 was increased and the expression of MDM2 was decreased (Figure 2A). And the MTT results showed zinc combined with Pmp53 increased the chemosensitivity of prostate cancer cells to PTX in both DU145 and PC3 cells (Figure 2B). We next examined the activity of ACO2 in DU145 and PC3 cells. ACO2 activity was significantly decreased after zinc or Pmp53 treatment compared with controls and further decreased in the zinc + Pmp53+PTX group (Figure 2C). Because ACO2 isomerizes citrate to isocitrate, we also examined the levels of both citrate and isocitrate in DU145 and PC3 cells. In the treatment groups with decreased ACO2 activity, citrate increased and isocitrate decreased (Figure 2D,E). These results suggest that increased chemosensitivity resulted from zinc and p53 is associated with decreased ACO2 activity in prostate cancer cells.

### 3.3 Increased ROS results in decreased ACO2 activity

**Figure 3:**
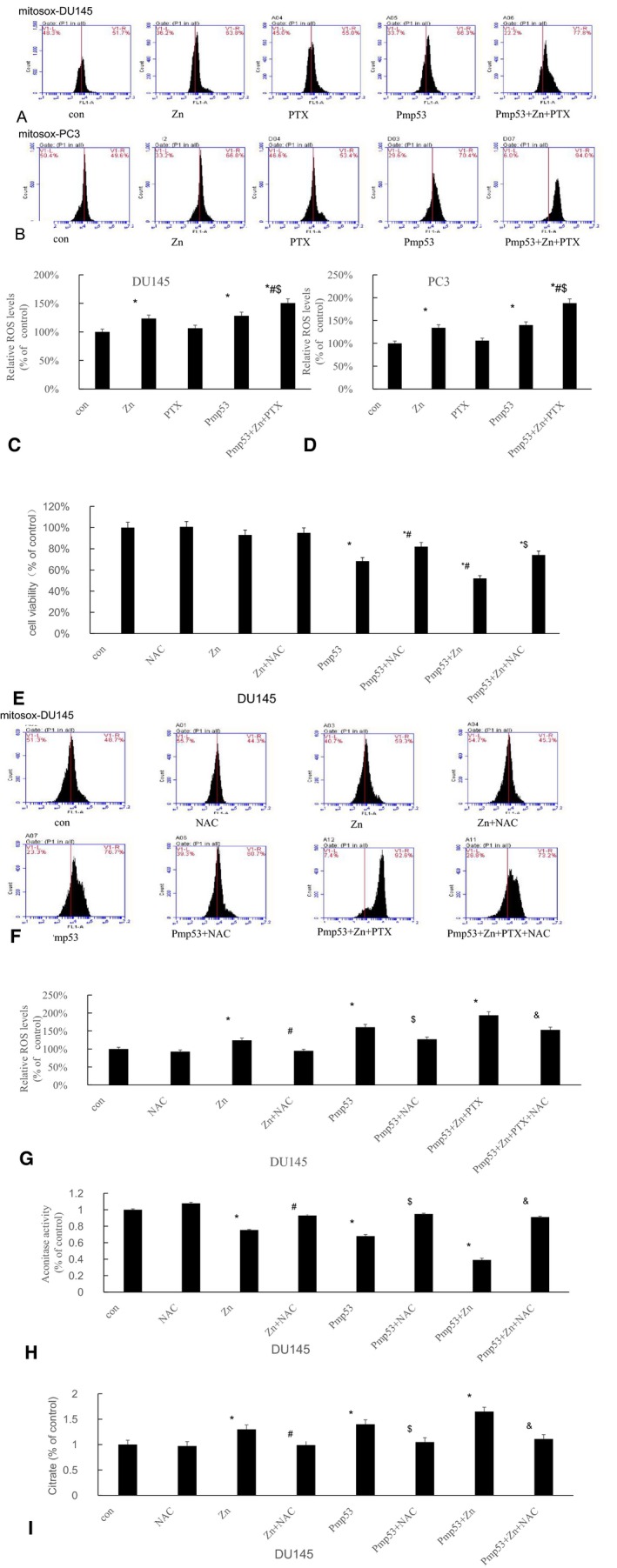
Increased reactive oxygen species (ROS) results in decreased ACO2 activity. (A, B) Treated DU145 and PC3 cells were stained with MitoSOX and flow cytometry was performed to evaluate ROS. (C, D) Statistical analysis of ROS levels in (A) and (B). *P < 0.01 vs control, #*P < *0.01 vs Zn, $*P < *0.01 vs Pmp53. (E) After different treatments, cell viability was determined by MTT assay. *P < 0.01 vs control, #*P* < 0.01 vs Zn, $*P* < 0.01 vs Pmp53. (F) DU145 cells were treated with NAC and stained with MitoSOX and flow cytometry was performed to evaluate ROS. (G) Statistical data of ROS levels in (F). *P < 0.01 vs control, #*P* < 0.01 vs Zn, $*P* < 0.01 vs Pmp53, &*P* < 0.01 vs Pmp53 + Zn + PTX. ACO2 activity (H) and citrate levels (I) were also detected. Bars, SD (n = 3). *P < 0.01 vs control, #*P* < 0.01 vs Zn, $*P* < 0.01 vs Pmp53, &*P* < 0.01 vs Pmp53 + Zn + PTX

Since ACO2 is sensitive to ROS, zinc and p53 have been reported to increase the level of ROS in cells. Therefore, we examined the effects of zinc and p53 on ROS and the effect of ROS levels on ACO2 activity. The result showed that in both DU145 and PC3 cells, ROS was significantly increased after zinc or Pmp53 treatment alone and further increased in the zinc + Pmp53 + PTX group (Figure 3A‐D). To further confirm the impact of ROS on cell survival, we added NAC, a scavenger of ROS, to treated cells and performed MTT assays. The results showed that the survival rate of cells was increased after treatment with NAC (Figure 3E). We also examined ROS levels after NAC treatment and found that NAC reverses the increase in ROS caused by zinc and p53 (Figure 3E,F). To confirm the effect of ROS on ACO2 activity, we next examined the relationship between ROS and ACO2 by evaluating ACO2 activity and citrate levels after NAC treatment. In cells treated with NAC, ACO2 activity was increased and citrate levels were decreased (Figure 3H,I). Together, these results indicate that zinc and p53 inhibit ACO2 activity by increasing ROS levels.

### 3.4 p53 decreases SIRT3 expression, leading to an increase in ROS and resulting in an decrease in ACO2 activity

**Figure 4:**
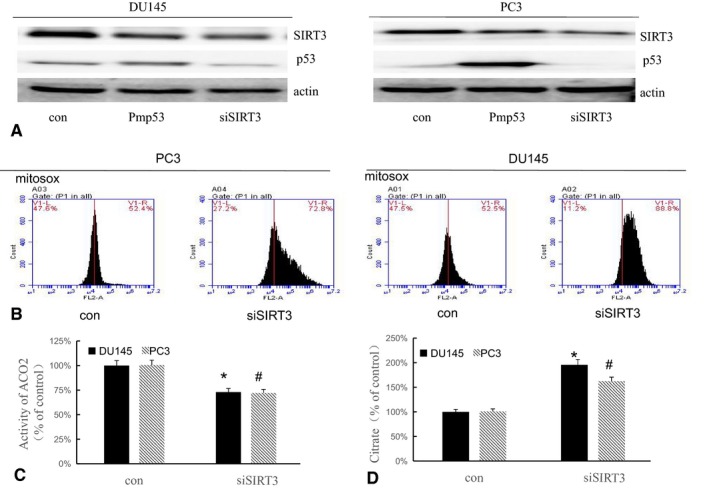
p53 decreases SIRT3 expression, leading to an increase in reactive oxygen species (ROS). (A) Western blot analysis for the expression of SIRT3 and p53 in DUI45 and PC3 cells after siSIRT3 or Pmp53 transfection. (B) DU145 and PC3 cells transfected with siSIRT3 were stained with MitoSOX and flow cytometry was evaluated ROS. ACO2 activity (C) and citrate levels (D) were detected after siSIRT3 transfection. Bars, SD (n = 3). *,#*P* < 0.01 vs control

Our results above showed that Pmp53 can lead to increased ROS in prostate cancer cells (Figure 3A,B). SIRT3 plays an important role in the regulation of oxidative stress and can be regulated by p53. Therefore, we first explored whether p53 regulates SIRT3 in prostate cancer cells by examining the expression of SIRT3 in cells transfected with Pmp53 (Figure 4A). The results showed that Pmp53 decreased SIRT3 expression. To confirm the regulation of SIRT3 on ROS levels and ACO2 activity in prostate cancer cells, we transfected DU145 and PC3 cells with siSIRT3 and evaluated ROS and citrate levels and ACO2 activity. The results showed that ROS levels were decreased in prostate cancer cells after transfected with siSIRT3 compared with controls (Figure 4B). We also found that after treatment with siSIRT3, ACO2 activity decreased, and citrate levels increased in DU145 and PC3 cells (Figure 4C). Together, these results suggest that p53 negatively regulates the levels of SIRT3, leading to increased ROS and decreased ACO2 activity in prostate cancer cells.

### 3.5 Zinc increases the production of hydroxyl radicals, leading to an increase in ROS and resulting in an decrease in ACO2 activity

**Figure 5:**
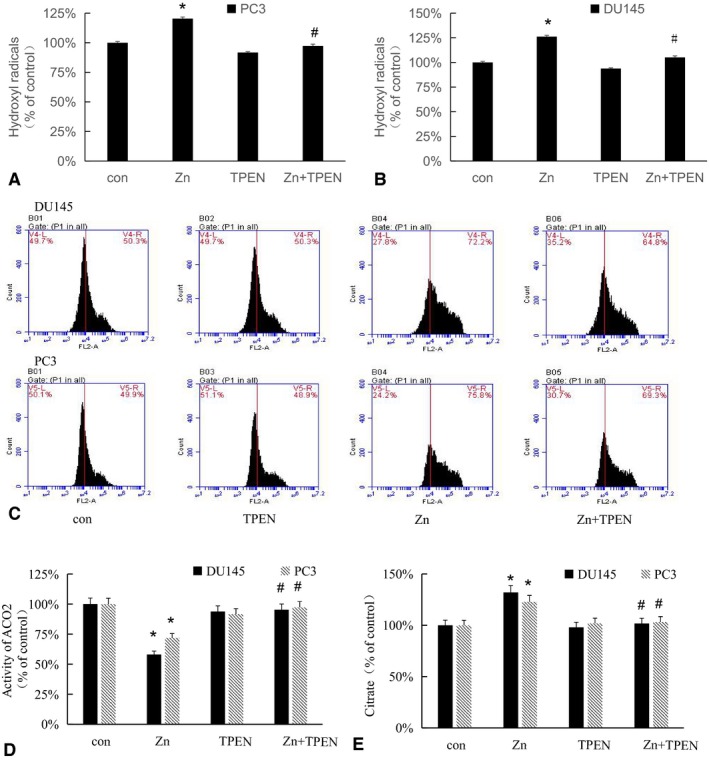
Zinc increases the production of hydroxyl radicals, leading to an increase in reactive oxygen species (ROS). Cells were treated with 200 µmol/L zinc and/or 10 nmol/L TPEN, and hydroxyl radicals (A, B), ROS (C), ACO2 activity (D) and citrate levels (E) were detected. Bars, SD (n = 3). *P < 0.01 vs control, #*P* < 0.01 vs Zn

Our results demonstrated that zinc leads to increased ROS in DU145 and PC3 cells (Figure 3A,B). We then measured hydroxyl radicals after zinc treatment, as these radicals are the main component of ROS. The results showed that hydroxyl radicals increased after zinc treatment (Figure 5A,B). To further explore the effect of hydroxyl radicals on ACO2 activity, we examined the levels of ROS, the activity of ACO2 and the levels of citrate. The results showed that ACO2 activity decreased and citrate accumulated along with the increase in ROS after zinc treatment (Figure 5C‐E). To further clarify the role of zinc, we treated DU145 and PC3 cells with TPEN, a zinc chelator, and the results showed that TPEN decreased hydroxyl radicals, ROS levels and citrate levels and partially recovered ACO2 activity (Figure 5A‐E). These results suggest that zinc can increase the production of hydroxyl radicals, which leads to an increase in ROS, thereby inhibiting the activity of ACO2.

### 3.6 Decrease in ACO2 activity leads to mitochondrial dysfunction

**Figure 6:**
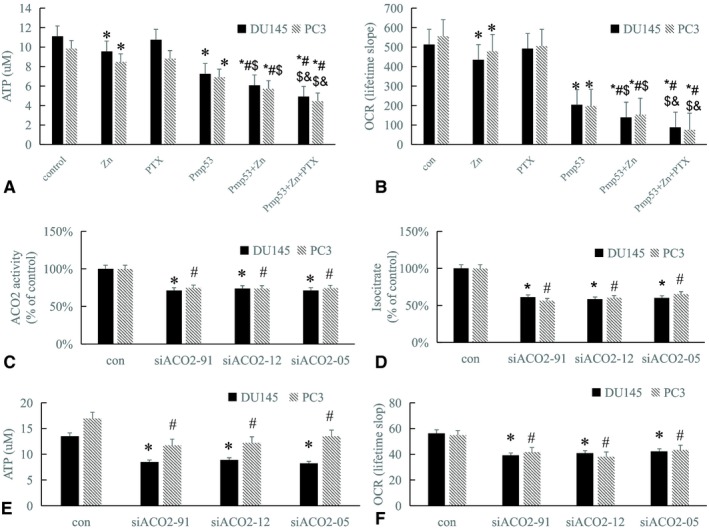
Decrease in ACO2 activity leads to mitochondrial dysfunction. ATP production (A) and oxygen consumption rates (OCR) (B) were measured after different treatments in DUI45 and PC3 cells. Bars, SD (n = 3). *P < 0.05 vs control, #*P* < 0.01 vs Zn, $*P* < 0.01 vs Pmp53, &*P* < 0.01 vs Pmp53 + Zn + PTX. ACO2 activity (C), isocitrate levels (D), ATP production (E) and OCR (F) were detected in DUI45 and PC3 cells after siAC2. Bars, SD (n = 3). *, #*P* < 0.01 vs control

ACO2 plays an important role in the maintenance of oxidative phosphorylation and energy production. Therefore, we next examined ATP levels and OCR to detect oxidative phosphorylation and energy production in DU145 and PC3 cells after zinc and/or Pmp53 treatment. The results showed that in cells transfected with Pmp53, ATP levels were significantly reduced compared with controls. Further reduction was observed when cells were treated with zinc and Pmp53 (Figure 6A), and oxidative phosphorylation was also significantly reduced compared with controls. Further reduction was also observed when cells were treated with zinc and Pmp53 (Figure 6B). To further explore the effects of ACO2 on mitochondrial function, we next evaluated oxidative phosphorylation by examining OCR and ATP levels after treatment with siACO2. The results showed that ACO2 activity and isocitrate were reduced after treatment with siACO2 (Figure 6C,D). Oxidative phosphorylation and ATP levels were significantly reduced in cells transfected with siACO2 compared with controls (Figure 6E,F). Together, these results suggest that the reduced activity of ACO2 caused by zinc and p53 can lead to mitochondrial dysfunction in prostate cells.

### 3.7 Mitochondrial dysfunction associated with ACO2 leads to apoptosis

**Figure 7:**
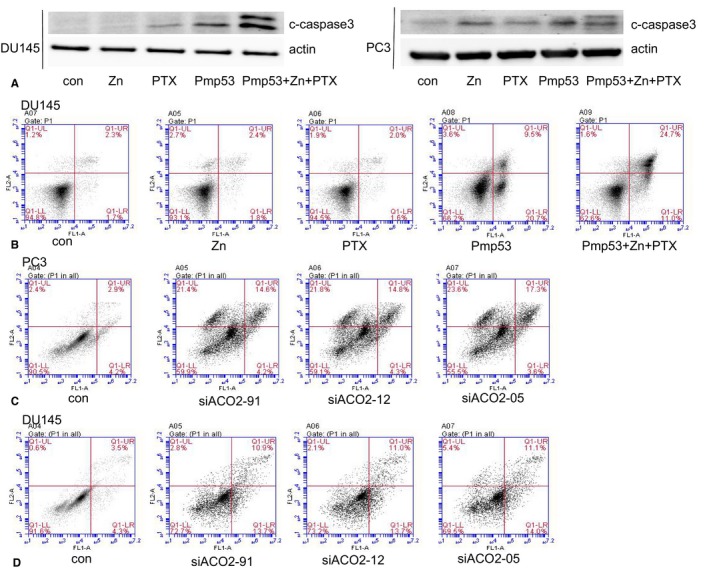
Mitochondrial dysfunction associated with ACO2 leads to apoptosis. DUI45 and PC3 cells were treated as indicated. The expression of cleaved‐caspase 3 was detected by western blot (A), and apoptosis rates were detected with Annexin V/PI staining and by flow cytometry (B). Apoptosis rates were detected with Annexin V/PI staining and flow cytometry in DUI45 and PC3 cells after siACO2 treatment (C, D)

The above results suggest that zinc combined with p53 may inhibit the Krebs cycle through ACO2, leading to dysfunction of mitochondria, which has been linked with apoptosis. We thus examined the expression of cleaved‐caspase 3 as a marker for apoptosis. Indeed, we found that cells treated with zinc and/or Pmp53 showed increased expression of cleaved‐caspase 3 (Figure 7A). We also detected apoptosis using Annexin‐V/PI. The results showed that the apoptosis rate of DU145 cells was significantly increased after treatment with zinc, Pmp53, and PTX (Figure 7B). To further clarify the effects of impaired ACO2 activity on apoptosis, we detected apoptosis after siACO2 treatment. The results show that siACO2 treatment can significantly induce apoptosis (Figure 7C,D). The results reveal that mitochondrial dysfunction associated with decreased ACO2 leads to apoptosis in prostate cancer cells.

### 3.8 Effects of zinc, Pmp53, PTX, and Pmp53 + zinc + PTX treatment in an in vivo tumor xenograft model

**Figure 8:**
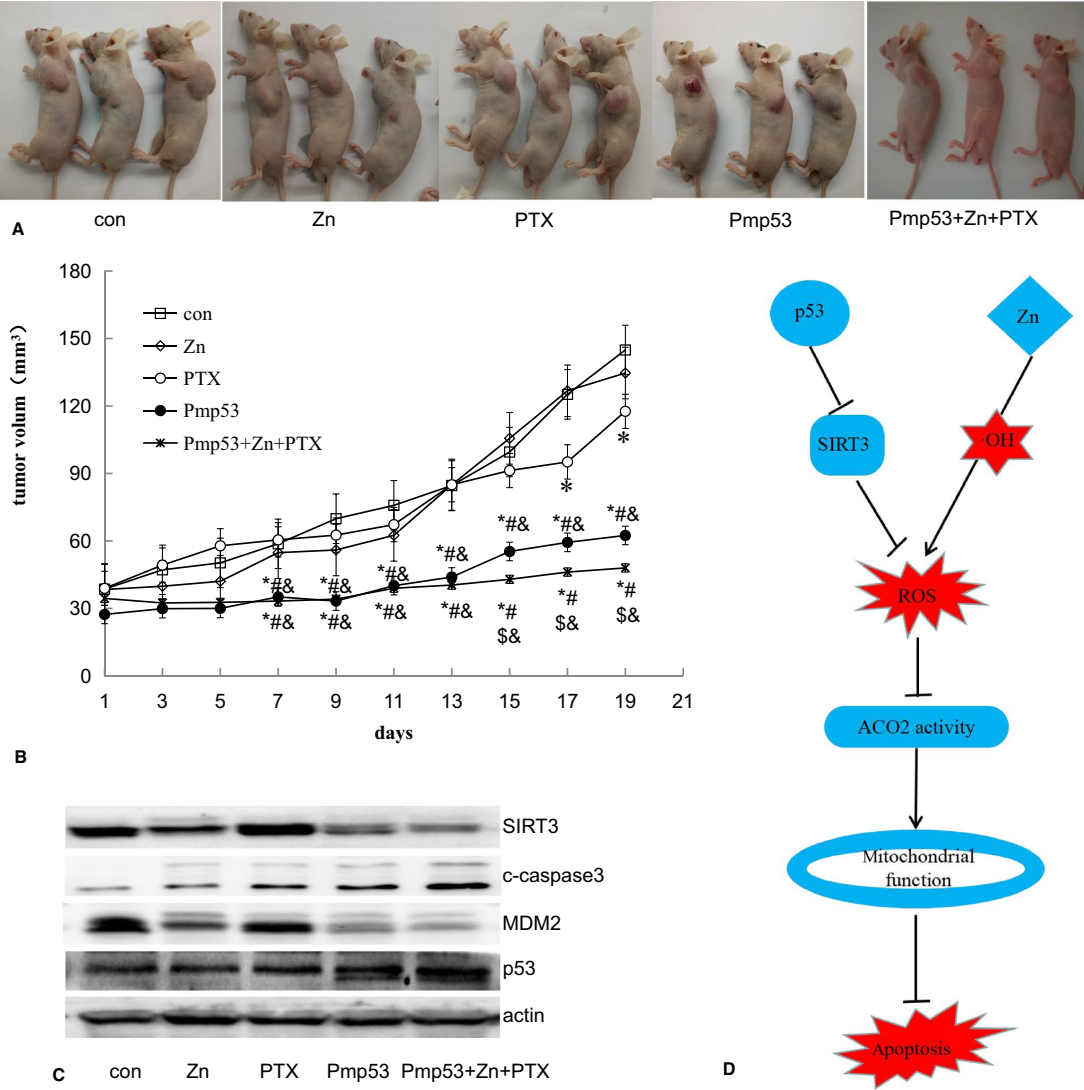
Effects of zinc, Pmp53, paclitaxel (PTX), and Pmp53 + zinc + PTX treatment in an in vivo tumor xenograft model. (A) Representative images of DU145 cell xenografts. DU145 cells were subcutaneously implanted into nude mice. Each treatment group consisted of three mice treated with zinc (10 mg/kg body weight), PTX (10 mg/kg body weight), and/or injected intravenously with attenuated *Salmonella typhi* Ty21a with Pmp53 plasmid for 20 days. (B) Tumor size was measured every 2 days and then tumor growth curves were generated. *P < 0.01 vs control, #*P* < 0.01 vs Zn, $*P* < 0.01 vs Pmp53, &*P* < 0.01 vs PTX (C) Western blot analysis for the expression of p53, MDM2, cleaved‐caspase 3, and SIRT3 after different treatments. (D) Zinc and p53 induce apoptosis in prostate cancer cells by inhibiting the activity of ACO2 through reactive oxygen species (ROS) accumulation. p53 decreases SIRT3 expression, leading to an increase in ROS. Zinc increases the production of hydroxyl radicals, leading to an increase in ROS. Increased ROS results in decreased ACO2 activity, which leads to mitochondrial dysfunction, followed by apoptosis

To evaluate the effect of treatment with zinc, Pmp53, PTX, and Pmp53 + zinc + PTX in vivo, we established a tumor xenograft model in nude mice. Pmp53 and Pmp53 + zinc + PTX treatments had significant inhibitory effects on tumors compared with controls, zinc, and PTX treatment (Figure 8A,B). And Pmp53 + zinc + PTX treatments have the most obvious inhibitory effect compared with other treatment groups, indicating a stronger effect with the combination treatment (Figure 8A,B). The expression of p53 and cleaved‐caspase 3 was significantly increased and MDM2 and SIRT3 were decreased in the Pmp53 and Pmp53 + zinc + PTX treatment groups compared with the controls (Figure 8C). These results were more pronounced in the Pmp53 + zinc + PTX‐treated group compared with the Pmp53 treatment group.

## 4 DISCUSSION

Cancers are characterized by altered metabolism, and metabolic enzymes, such as ACO2, that show significant differences between cancer and normal cells are attractive molecular targets for cancer treatment. The ACO2 metabolic enzyme has been associated with the development of many diseases, including cancer.^10^, ^12^, ^16^, ^17^, ^34^ We investigated ACO2 protein expression in prostate cancer T and ANT tissues. Here we show, for the first time, that the expression of ACO2 was significantly increased in prostate cancer T tissues compared with ANT tissues. We also identified a positive correlation between ACO2 expression and the malignancy of prostate cancer. We then found that the protein expression and activity of ACO2 were higher in PTX‐resistant PC3 cells than in PTX‐sensitive DU145 cells. Our results indicate that ACO2 plays an important role in the development and drug resistance of prostate cancer and suggests that targeting ACO2 may be a useful strategy for prostate cancer treatment.

Previous studies showed that the activity of ACO2 is regulated by ROS levels.^10^ Tórtora found that nitric oxide, S‐nitrosoglutathione, and peroxynitrite can inactivate highly purified recombinant ACO2.^15^ Reduced ACO2 activity has been observed in some neurodegenerative lesions and senescence associated with oxidative stress.^27^, ^28^ We also found that an increase in ROS was accompanied with a decrease in ACO2 activity in PC3 and DU145 cells and showed that NAC can reduce ROS levels and restore ACO2 activity in prostate cancer cells. Together, these results demonstrate that ACO2 activity is regulated by ROS.

Zinc is significantly reduced in prostate cancer tissue and negatively correlated with prostate cancer progression.^5^, ^35^, ^36^ Provinciali and Heim found that zinc can increase intracellular ROS.^19^, ^37^ In addition, Guo found that zinc induced the production of hydroxyl radicals and led to apoptosis in rat retinal ganglion cells.^20^ We also found that exposure of prostate cancer cells to 200 µmol/L zinc for 24 hours could increase hydroxyl radicals and decrease the activity of ACO2. TPEN, an inhibitor of zinc, reduced hydroxyl radicals and restored ACO2 activity. Our results show that zinc‐induced ROS production plays an important role in the inactivation of ACO2.

SIRT3 is a key regulator of oxidative stress that regulates the transcription and deacetylation of SOD2 to clear ROS.^27^, ^28^ Several studies have demonstrated a close relationship between p53 and SIRT3. Li et al found that p53 could regulate SIRT3 deacetylase activity and induce growth inhibition.^31^ However, the relationship between p53 and SIRT3 in cancer is not yet clear. We found that overexpression of p53 reduced the expression of SIRT3, which led to an increase in ROS. In addition, siSIRT3 led to increased ROS and reduced ACO2 activity in prostate cancer cells. Therefore, we speculate that the p53 may act as a transcription factor that inhibits SIRT3, leading to increased ROS and impaired ACO2 activity in prostate cancer cells.

Our results showed that inhibition of ACO2 activity in prostate cancer cells could lead to impaired oxidative phosphorylation and production. Cheng found that glycolysis and Krebs cycle were significantly impaired and ATP was reduced in a Drosophila model of mitochondrial aconitase deficiency.^11^ Abela performed plasma metabolomics analysis of patients with ACO2 deficiency and showed that Krebs cycle metabolites were significantly reduced in these patients.^38^ Taken together, these data suggest that ACO2 plays an important role in the maintenance of mitochondrial function.

In summary, here we show that ACO2 is overexpressed in malignant prostate cancer tissues compared with ANT tissues, and its expression is positively correlated with the malignancy of prostate cancer. Furthermore, ACO2 expression is involved in prostate cancer drug resistance. In addition, we found that both zinc and p53 lead to an increase in ROS. p53 decreases SIRT3 expression, leading to an increase in ROS, while zinc increases the production of hydroxyl radicals, leading to an increase in ROS. Accumulated ROS then causes a decrease in ACO2 activity, and impaired ACO2 activity leads to mitochondrial dysfunction, resulting in apoptosis (Figure 8D). Our in vivo experiments found that zinc combined with p53 can significantly increase the antitumor effect of PTX. Thus, ACO2 can be a target for remodeling metabolism by sensing changes in ROS of prostate cancer cells. Targeting ACO2 through zinc and p53 to change the metabolic pattern of malignant cells may provide a new therapeutic strategy for prostate cancer.

## CONFLICT OF INTEREST

The authors have no conflict of interest.
